# Forensic age assessment by 3.0 T MRI of the wrist: adaption of the Vieth classification

**DOI:** 10.1007/s00330-022-08819-y

**Published:** 2022-05-04

**Authors:** C. Ottow, S. Schmidt, W. Heindel, H. Pfeiffer, B. Buerke, A. Schmeling, V. Vieth

**Affiliations:** 1 Clinic for Radiology, University of Münster and University Hospital Münster, Albert-Schweitzer-Campus 1, 48149 Münster, Germany; 2 Institute of Legal Medicine, University of Münster and University Hospital Münster, Röntgenstraße 23, 48149 Münster, Germany; 3 Clinic for Radiology, University of Münster and University Hospital Münster, Albert-Schweitzer-Campus 1, 48149 Münster, Germany; 4 Present Address: Clinic for Radiology, Klinikum Ibbenbüren, Große Straße 41, 49477 Ibbenbüren, Germany

**Keywords:** Wrist, Magnetic resonance imaging, Radius, Ulna, Age determination by skeleton

## Abstract

**Objectives:**

In order to find a reliable method to correctly assess majority in both sexes by MRI, a study was conducted to evaluate the applicability of the recently presented Vieth classification in wrist MRI, after it had originally been proposed for knee MRI.

**Methods:**

After receiving a positive vote by the ethics committee, the left-hand wrists of 347 male and 348 female volunteers of German nationality in the age bracket 12–24 years were scanned. Before conducting the prospective, cross-sectional examinations, an informed consent was obtained from each volunteer. A 3.0 T MRI scanner was used, acquiring a T1 turbo spin-echo sequence (TSE) and a T2 TSE sequence with fat suppression by spectral presaturation with inversion recovery (SPIR). The images were assessed by applying the Vieth classification. Minimum, maximum, mean ± standard deviation, and median with lower and upper quartiles were defined. Intra- and interobserver agreements were determined by calculating the kappa coefficients. Differences between the sexes were analyzed using the Mann-Whitney *U* test.

**Results:**

By applying the unmodified Vieth classification with corresponding schematics, it was possible to assess majority in both sexes via the epiphyseal-diaphyseal fusion of the distal radius and in males also via the epiphyseal-diaphyseal fusion of the distal ulna. The Mann-Whitney *U* test implied significant sex-related differences for all stages. For both epiphyses, the intra- and interobserver agreement levels were very good (*κ* > 0.8).

**Conclusion:**

If confirmed by further studies, it would be possible to determine the completion of the 18th year of life in both sexes by 3.0 T MRI of the wrist and using the Vieth classification.

**Key Points:**

*• The Vieth classification allows determining majority in males and females alike based on the distal radius’ epiphysis by 3.0 T MRI of the wrist.*

*• The Vieth classification also allows determining majority in males based on the distal ulna’s epiphysis by 3.0 T MRI of the wrist, but not in females.*

*• The presented data can be deemed referential within certain discussed boundaries.*

## Introduction

Along with the constant influx of individuals without proper personal data into European countries arises the need for dispassionate methods to determine majority, which is attained by the completion of the 18th year of life in most European countries [1].

In Germany, the Study Group on Forensic Age Diagnostics (AGFAD) issued recommendations for forensic age estimation in 2001 that were updated in 2008.

In judicial proceedings, in which it is to be proved with the highest level of certainty that the legally relevant age threshold is surpassed, the AGFAD’s approach follows the “minimum-age concept” [2]. This means that the chronological age of the youngest individual of the referential study who shows the same developmental stage as the examined individual is assumed to be the individual’s minimum age. Though this method does not provide a precise actual age, it will almost always lead to a lower estimated age compared to the actual age and should prevent an overestimation of the chronological age and subsequently a falsely attested majority. Therefore, minimum ages are of the utmost importance in applied forensic age assessment.

In practice, this method represents the only reliable way to reproducibly determine whether an individual has attained majority, but also means that ionizing radiation needs to be applied since it relies on projectional radiography, orthopantomograms, and case-dependent also computed tomographies. However, the use of ionizing radiation without medical indication and outside of criminal investigations is not uncontroversial. Since the individuals in question are rather subject to proceedings of registration offices and not to criminal investigations, recent research in this field has concentrated on finding additional reliable methods, free of ionizing radiation.

Lately, while also expanding research on already known techniques [3–8], a multitude of different approaches and modalities have been tried [9–28], but to some extent failed to meet the AGFAD’s requirements [29]. With the presented new classification of Vieth et al [30], we could finally establish a reliable method for the evaluation of long bones’ epiphyses, free of ionizing radiation. The classification was originally formulated for evaluating the epiphyseal-diaphyseal fusion of the knee joint’s epiphyses, namely those of the distal femur’s and the proximal tibia’s epiphyses. However, Vieth et al themselves already suggested that the classification is likely to be adaptable to the epiphyses of other long bones as well [2]. This could in turn provide additional possibilities to correctly assess majority in one or both sexes. Since it is known that the timeframes of ossification and fusion differ in the upper and lower parts of the appendicular skeleton as well as in its proximal and distal parts, this would likely show different minimum ages of the stages. The present study takes these assumptions up by modifying, applying, and evaluating the Vieth classification concerning the distal long bones’ epiphyses of the wrist.

## Materials and methods

The study uses a dataset that was acquired by the European study “Age estimation in unaccompanied minors by means of MRI.” Multiple regions of interest of several hundreds of volunteers were scanned and are currently being analyzed. Due to this, parts of the cohort’s results have already been reported concerning different approaches or other regions of interest.

After the responsible ethics committee had voted in favor of the study, the recruitment of the volunteers via advertisement in local media and the internet was begun. Each of the volunteers or their legal guardian, in case of minors, had signed a written consent prior to their examination.

Between May 2013 and June 2015, 347 male and 348 female volunteers in the age bracket 12–24 years of German nationality were prospectively scanned (see Table 1). Noted characteristics of the volunteers included sex, proven age, and known illnesses as well as past and present medication. The participants were to be distributed evenly across groups of up to 25 per year of age and sex. Exclusion criteria for the recruitment and the examination were the presence of skeletal development–relevant diseases and/or disorders, traumata to the knee joint area, and the common contra-indications of magnetic resonance imaging, especially incorporated metal elements, recent surgical procedures, freshly implanted vascular clips, claustrophobia, and potential gravidity. The wrist was chosen as the region of interest (ROI), since earlier studies suggested its epiphyses to possibly show relevant morphological changes around the 18th year of life in both sexes [28, 31].

**Table 1:** Case group figures (*n* = 695)

Chronological age (in years)	Male volunteers	Female volunteers
12	21	16
13	30	14
14	20	27
15	25	29
16	26	31
17	28	28
18	29	29
19	26	31
20	29	29
21	32	31
22	27	30
23	27	29
24	27	24
	Σ 347	Σ 348

The MRI scans were performed on a 3.0 T scanner (Philips 3.0 T Achieva, gradients 80 mT/m; Philips Medical Systems) using a high-resolution surface coil (Sense Flex M). The scans were to be repeated in case of movement artifacts.

A T1-weighted turbo spin-echo (T1 TSE) sequence in coronal orientation was used (TR shortest; TE 11 ms; flip angle 90; duration 3:33 min; measured voxel size 0.4 × 0.5 × 2.5 mm; reconstructed voxel size 0.2 × 0.2 × 2.5 mm). Furthermore, an additional T2-weighted turbo spin-echo sequence with signal presaturation with inversion recovery (T2 TSE SPIR) also in coronal orientation was used (TR range 3000–4000 ms; TE 70 ms; flip angle 90; duration 3:36 min; measured voxel size 0.4 × 0.51 × 2.5 mm; reconstructed voxel size 0.2 × 0.2 × 2.5 mm).

The images were viewed on a PACS workstation and first evaluated by an examiner with more than 8 years of experience in musculoskeletal MRI diagnostics and age estimation (C.O.). To determine the intraobserver agreement, a re-evaluation of 100 randomly chosen cases was performed after a lapse of 2 months so as to prevent recall bias. A second examiner with more than 20 years of experience in musculoskeletal MRI diagnostics and age estimation (V.V.) also evaluated the same group of 100 cases for determining the interobserver agreement. The evaluations were performed without knowledge of sex, age, and earlier evaluations of the examined volunteers.

Statistical analyses were conducted using IBM SPSS Statistics 24 (Build 1.0.0.407) for Mac OS X (release 15/03/2016, IBM Corporation). Minimum, maximum, mean ± standard deviation, and median with lower and upper quartiles were defined for each stage of the classification to find the minimal ages. Intra- and interobserver agreements were determined by calculating the unweighted kappa coefficients. Sex-related differences in the stage assessment across the ages were analyzed using the Mann-Whitney *U* test to determine their statistical relevance (*p* < 0.05, exact, two-tailed).

## Results

The examination of the distal radius’ epiphysis and the distal ulna’s epiphysis was possible in all cases, using the sequences that had originally been described for the knee joint by Vieth et al.

### Cohort

We prospectively scanned and evaluated 695 volunteers, ranging from 12.05 to 25.00 years of age. For male volunteers, the median age at the point of examination was 18.83 years, while the median age for female volunteers was 19.01 years.

### Sequencing

A T1 TSE sequence was utilized to acquire images aiming for osseous structures. An additional T2 TSE SPIR sequence was utilized to acquire images aiming for stationary watery components within the previously depicted osseous structures and/or in cartilaginous parts. The examinations approximately took 10 to 15 min to complete, including positioning.

### MRI classification

The examinations of the volunteers were sorted separately for both sexes from youngest to oldest, while blinding the exact age. All slices of each examination were looked through to get an overview of the general morphology of the growth plate and the process of its ossification. Initial sketches of the anatomical features and changes of the growth plate were then drawn for both sequences. Only those features that could be observed in most cases of similar age were taken into account. As the morphological appearance of the growth plate showed no sex-related differences, the separation by sex was dropped.

**Fig. 1:**
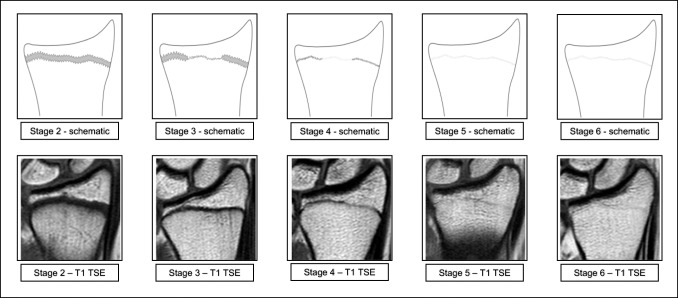
Schematic drawings for the stages of the T1 TSE sequence and examples (3.0 T; non-contrast enhanced; coronal slice orientation); from left to right: female 12 years, female 14 years, female 16 years, male 18 years, female 24 years. **Abbreviations**: *MRI*, magnetic resonance imaging; *TSE*, turbo spin-echo. Please note: Stage 5 and stage 6 are identical in the T1 TSE sequence and can only be further differentiated by the additional acquisition of the T2 TSE SPIR sequence

**Fig. 2:**
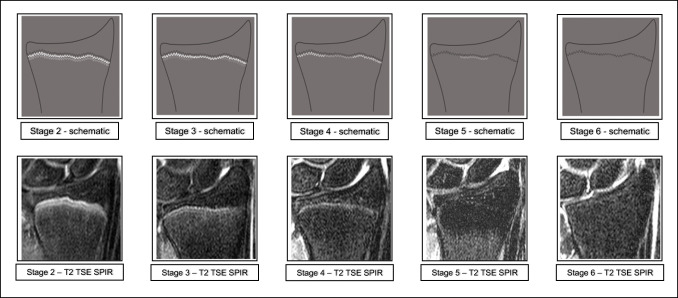
Schematic drawings for the stages of the T2 TSE SPIR sequence and examples (3.0 T; non-contrast enhanced; coronal slice orientation); from left to right: female 12 years, female 14 years, female 16 years, male 18 years, female 24 years. **Abbreviations**: *MRI*, magnetic resonance imaging; *TSE*, turbo spin-echo; *SPIR*, signal presaturation with inversion recovery

Early on, it became obvious that the developmental continuum originally described by Vieth et al [30] was clearly identifiable in the distal radius’ epiphysis and the distal ulna’s epiphysis as well. No further modification beyond this had to be made on the classification. Accordingly, the MRI classification of Vieth et al was then applied with adjusted schematics for the epiphyses of interest. The classification is centered around the acquisition of an anatomically reliable T1 TSE sequence that also provides information about the osseous epiphyseal-diaphyseal fusion and the additional acquisition of a T2 TSE SPIR sequence with which to locate watery components. The latter provides the critical information needed for differentiation of the final stages, but lacks the anatomical precision of the T1 TSE sequence that is essential in locating the exact course of the growth plate or its scar after the finished fusion and allows the discrimination of other structures (see Figs. 1 and 2 for the corresponding schematics and examples; see Table 2 for the shortened version):

**Table 2:** MRI classification as according to Vieth et al

Stage	Sequences	Characteristics of the epiphyseal ossification
2	T1-TSE and T2-TSE SPIR	Continuous intermediate band in T1-TSE and two continuous or discontinuous hyperintense lines in T2-TSE SPIR
3	T1-TSE and T2-TSE SPIR	Discontinuous intermediate band in T1-TSE and two hyperintense, sporadically convening lines in T2-TSE SPIR
4	T1-TSE and T2-TSE SPIR	Discontinuous intermediate line in T1-TSE and a thin discontinuous or dotted hyperintense line in T2-TSE SPIR
5	T1-TSE and T2-TSE SPIR	Continuous intermediate line in T1-TSE and discontinuous hyperintense line in T2-TSE SPIR
6	T1-TSE and T2-TSE SPIR	Continuous intermediate line in T1-TSE and no signal in T2-TSE SPIR in the same position

**Abbreviations**: *MRI*, magnetic resonance imaging; *TSE*, turbo spin-echo; *SPIR*, signal presaturation with inversion recovery

Stage 2 In the T1 TSE sequence, a continuous band of intermediate signal intensity is visible, walled by serrated lines of low to none signal intensity towards the epiphysis and the diaphysis.

In the T2 TSE SPIR sequence, the epiphysis is demarked by a serrated line of low to none signal intensity. The metaphysis shows two serrated lines of high signal intensity. Both lines can be continuous or discontinuous.

Stage 3 In the T1 TSE sequence, a discontinuous band of intermediate signal intensity is visible. The band is walled by serrated lines of low to none signal intensity towards the epiphysis and the diaphysis that sporadically convene and interrupt the band, forming a single serrated line with no signal intensity.

In the T2 TSE SPIR sequence, the metaphysis shows two serrated lines of high signal intensity that sporadically convene, forming a single thin and serrated line of high signal intensity.

Stage 4 In the T1 TSE sequence, a discontinuous thin and serrated line of intermediate signal intensity between the epiphysis and the diaphysis is visible. In the continuity of the line, thicker sections with no signal intensity can be seen.

In the T2 TSE SPIR sequence, a thin single, discontinuous or dotted line of hyperintense signal is visible in the same position as the described thin line of the corresponding T1 TSE sequence. In the continuity of the line, thicker hyperintense sections can be seen.

Stage 5 In the T1 TSE sequence, a continuous thin line of intermediate signal intensity between the epiphysis and the diaphysis is visible.

The T2 TSE SPIR sequence shows a single thin, discontinuous, or dotted line of hyperintense signal in the same position as the described thin line of the corresponding T1 TSE sequence.

Stage 6 In the T1 TSE sequence, a continuous thin line of intermediate signal intensity between the epiphysis and the diaphysis is visible.

The T2 TSE SPIR sequence shows no hyperintense signal in the same position as the described thin line of the corresponding T1 TSE sequence.

The stages are based on the presence or absence of certain landmarks of the epiphyseal-diaphyseal fusion’s morphological appearance, mainly drawn from the T1 TSE sequence. In the T1 TSE sequence, they are characterized by the presence of a continuous band-like morphology of the growth plate (stage 2), the presence of a discontinuous band-like morphology (stage 3), the beginning thin-lined demarcation of the fusion’s “scar” (stage 4), the completely demarcated fusion’s “scar” with a corresponding T2 TSE SPIR-hyperintense signal (stage 5), and finally without a corresponding T2 TSE SPIR-hyperintense signal (stage 6). Stages 5 and 6 have the exact same appearance in the T1 TSE sequence and cannot be differentiated without the T2 TSE SPIR sequence.

Applying this classification, it was possible to assess a respective stage of the distal radius’ epiphysis and the distal ulna’s epiphysis in all cases (695). The evaluation took below 1 min per case.

### Observed minimum ages in the distal radial epiphysis

In male individuals, the minimum ages for stages 2, 3, 4, 5, and 6 of the epiphyseal-diaphyseal fusion of the distal radius’ epiphysis were 12.05, 13.69, 15.35, 16.59, and 19.19 years respectively (compare Table 3).

**Table 3:** Relevant age values of the distal radius’ epiphysis ossification stages for male volunteers (in years)

Stage	Number of cases	Minimum	Maximum	Mean value; standard deviation	Lower quartile; median; upper quartile
2	67	12.05	17.07	13.63; 1.18	12.63; 13.59; 14.03
3	54	13.69	19.15	15.99; 1.25	15.07; 15.92; 16.76
4	49	15.35	20.94	17.94; 1.30	17.11; 17.79; 18.51
5	117	16.59	24.96	21.22; 1.92	19.57; 21.14; 22.71
6	60	19.19	24.98	22.74; 1.63	21.32; 23.16; 24.19

Please note: Values close to 12 years of life, to 24 years of life and deduced data of those values are presumably affected by the cohort’s age range

In female individuals, the minimum ages for stages 2, 3, 4, 5, and 6 of the epiphyseal-diaphyseal fusion of the distal radius’ epiphysis were 12.11, 12.30, 14.44, 15.94, and 18.86 years respectively (compare Table 4).

**Table 4:** Relevant age values of the distal radius’ epiphysis ossification stages for female volunteers (in years)

Stage	Number of cases	Minimum	Maximum	Mean value; standard deviation	Lower quartile; median; upper quartile
2	15	12.11	13.75	12.73; 0.52	12.25; 12.56; 13.23
3	56	12.30	17.82	14.67; 1.14	13.88; 14.73; 15.40
4	80	14.44	23.98	17.41; 2.05	15.98; 16.87; 18.48
5	130	15.94	25.00	20.62; 2.25	18.89; 20.24; 22.46
6	67	18.86	24.74	22.32; 1.51	21.19; 22.39; 23.65

Please note: Values close to 12 years of life, to 24 years of life and deduced data of those values are presumably affected by the cohort’s age range

### Observed minimum ages in the distal ulnar epiphysis

In male volunteers, the minimum ages for stages 2, 3, 4, 5, and 6 of the epiphyseal-diaphyseal fusion of the distal ulna’s epiphysis were 12.05, 14.14, 15.18, 16.59, and 18.50 years respectively (compare Table 5).

**Table 5:** Ossification stages of the distal ulna’s epiphysis in relation to age for male volunteers (in years)

Stage	Number of cases	Minimum	Maximum	Mean value; standard deviation	Lower quartile; median; upper quartile
2	72	12.05	17.07	13.72; 1.20	12.68; 13.66; 14.23
3	51	14.14	19.15	16.20; 1.27	15.25; 16.20; 16.97
4	38	15.18	20.94	17.73; 1.24	16.80; 17.64; 18.44
5	103	16.59	24.96	21.20; 2.16	19.26; 21.52; 22.75
6	83	18.50	24.98	22.08; 1.79	20.62; 22.03; 23.56

Please note: The values bordering to 12 years of life and to 24 years of life as well as derived data of those values are likely influenced by the cohort’s age range

In female volunteers, the minimum ages for stages 2, 3, 4, 5, and 6 of the epiphyseal-diaphyseal fusion of the distal ulna’s epiphysis were 12.11, 12.30, 14.44, 15.49, and 17.49 years respectively (compare Table 6).

**Table 6:** Relevant age values of the distal ulna’s epiphysis ossification stages for female volunteers (in years)

Stage	Number of cases	Minimum	Maximum	Mean value; standard deviation	Lower quartile; median; upper quartile
2	17	12.11	13.75	12.76; 0.54	12.89; 12.56; 13.23
3	60	12.30	17.82	14.82; 1.11	14.14; 14.75; 15.49
4	64	14.44	21.40	17.31; 1.69	15.99; 17.03; 18.48
5	91	15.94	25.00	20.20; 2.46	18.03; 19.95; 22.42
6	116	17.49	24.91	21.80; 1.85	20.43; 21.73; 23.45

Please note: Values close to 12 years of life, to 24 years of life and deduced data of those values are presumably affected by the cohort’s age range

### Intra- and interobserver agreement

After calculating Cohen’s kappa, we found a very good intraobserver-agreement level concerning the distal radius’ epiphysis (*κ* = 0.885) and the distal ulna’s epiphysis (*κ* = 0.850).

After calculating Cohen’s kappa, we found a very good interobserver-agreement level concerning the distal radius’ epiphysis (*κ* = 0.872) and the distal ulna’s epiphysis (*κ* = 0.813).

### Statistical differences of the sexes

The performed Mann-Whitney *U* test concerning the distal radius’ epiphysis implies significant sex-related differences for all stages: stage 2 (*p* < 0.01), stage 3 (*p* < 0.01), stage 4 (*p* < 0.01), stage 5 (*p* < 0.01), stage 6 (*p* < 0.01).

The performed Mann-Whitney *U* test concerning the distal ulna’s epiphysis implies significant sex-related differences for all stages: stage 2 (*p* < 0.01), stage 3 (*p* < 0.01), stage 4 (*p* < 0.01), stage 5 (*p* < 0.01), stage 6 (*p* < 0.01).

## Discussion

When applying the Vieth classification to the long bones’ epiphyses of the wrist, it is possible to determine the completion of the 18th year of life.

Majority can be concluded from a documented final stage in both sexes. In male individuals, the earliest stage 6 was found at 19.19 years in the distal radius’ epiphysis and at 18.5 years in the distal ulna’s epiphysis. In female individuals, the earliest stage 6 of the distal radius’ epiphysis was found at 18.86 years and at 17.49 years in the distal ulna’s epiphysis. This means that a stage 6 of either epiphysis can determine majority in males, while only a stage 6 of the distal radius’ epiphysis can determine majority in females.

However, the results are subject to bias and inherently limited. The stages’ results showing a minimum age close to 12 years of life and those showing a maximum age of 24 years of life are most likely influenced by the chosen age range (12–24 years of life) and are therefore the product of a selection bias. This also influences all derived numbers of those stages. Nonetheless, the recommended minimum age concept does not rely on these artificial values, but solely on the minimum age of the respective stage. This means that the documented minimum age for the stages 3–6 of the radius and the ulna in males and those for the stages 3–6 of the radius and the ulna in females can reliably be used for forensic age assessment.

In concordance with similar studies [17, 18, 25, 28, 32, 33], female individuals displayed an earlier start and a faster progression of the epiphyseal-diaphyseal fusion. Additionally, the distal ulna’s epiphysis finishes the fusion earlier when compared to the distal radius’ epiphysis.

Furthermore, when relating the results to those of Vieth et al concerning the staging of the knee joint’s epiphyses, we can find additional implications. The distal epiphyses of the radius and the ulna in our study completed the epiphyseal-diaphyseal fusion at an earlier age than the distal femur’s and the proximal tibia’s epiphyses in the study by Vieth et al. Their results showed only few cases of the final stage and Vieth et al stated that these would most likely represent the lower extremes of stage 6. The present study’s results support this assumption, as the earlier finished fusion of the distal radius’ and distal ulna’s epiphyses comes with a higher count of the final stages in both sexes.

We also need to take a look at other MRI classifications for the purpose of forensic age assessment. The most prominent one from Dvorak et al [20, 21] uses a T1w spin echo (SE) sequence and is thereby in parts comparable to the T1 TSE sequence of the present study. The results of Dvorak et al show a minimum age of their final stage (grade VI) in the age bracket of 16–17 years of life in male individuals. This stage is morphologically similar to stage 5 of the Vieth classification and its minimum age is comparable. However, it is for this very reason not suited for the minimum age concept since the highest obtainable minimum age of the Dvorak classification lies below 18.0 years of life.

Due to the usage of the common dataset from the European study “Age estimation in unaccompanied minors by means of MRI,” we can directly compare the current results with those of a study by Timme et al [34], who published results following a different approach, but concerning the same cohort. They sifted the images of the distal wrist for the threefold stratification of the epiphyseal scar, a distinct morphological feature, which had initially been described by Schmidt et al [31]. It is characterized by a pattern of two T1w hypointense metaphyseal lines with a T1w hyperintense band in between, running parallel to the epiphysis within the continuity of the former fusion zone after the concluded epiphyseal-diaphyseal fusion. Timme et al reported a minimum age of 18.6 years in male volunteers and 16.8 years in female volunteers, thereby making it a potential indicator of maturity in males, though the low margin of 0.6 years warrants caution. Since the sequence used by Schmidt et al and Timme et al is the same T1w TSE sequence as the one used for the Vieth classification, a cross comparison of the results should be conducted on the cohort. When applied in conjunction, a beneficial effect appears plausible, but further research is needed. Additionally, the number of participants of the studies differs since we did not have to exclude volunteers due to motion artifacts (current 695 vs. prior 668 cases). This most likely stems from a higher proneness of the depiction of the trabecular structures composing the threefold stratification sign towards blurring through movement and implies a more robust approach by Vieth et al.

The use of the presented findings is limited to examinations performed with 3.0 T scanners, as the field strength is likely to influence the examinations’ results towards finished fusion stages. Though the study’s cohort consisted only of German volunteers, this does not limit the applicability of the results to individuals of German nationality. As Schmeling et al have shown [35, 36], the major limiting factor in skeletal maturation is the socioeconomical status, while the Western Caucasian ethnicity shows the fastest progression of the skeletal development. This means that the findings will most likely lead to a lower stage assessment in favor of individuals of a lower socioeconomical status and/or different ethnicities.

Lastly, the study’s findings should be tried, tested, and verified by other study groups before the implementation, ideally by further prospective studies with a sufficient number of participants.

## Abbreviations

MRI: Magnetic resonance imaging
SPIR: Signal presaturation with inversion recovery
T1w: T1-weighted
T2w: T2-weighted
TSE: Turbo spin-echo

## Declarations

### Guarantor

The scientific guarantor of this publication is Professor Andreas Schmeling.

### Statistics and biometry

No complex statistical methods were necessary for this paper.

### Study subjects or cohorts overlap

Some study subjects or cohorts have been previously reported in:

Ottow C, Krämer JA, Olze A, et al (2014) Magnetresonanztomographiestudie zur Altersschätzung von unbe-gleiteten minderjährigen Flüchtlingen [Magnetic resonance tomography studies on age estimation of unaccompanied minor refugees]. Rechtsmedizin 25(1):12-20

Guo Y, Olze A, Ottow C, et al (2015) Dental age estimation in living individuals using 3.0 T MRI of lower third molars. Int J Legal Med 129:1265-1270

Ottow C, Schulz R, Pfeiffer H, Heindel W, Schmeling A, Vieth V (2017) Forensic age estimation by magnetic resonance imaging of the knee: the definite relevance in bony fusion of the distal femoral- and the proximal tibial epiphyses using closest-to-bone T1 TSE sequence. Eur Radiol 27:5041-5048

Timme M, Ottow C, Schulz R, et al (2017) Magnetic resonance imaging of the distal radial epiphysis: a new criterion of maturity for determining whether the age of 18 has been completed? Int J Legal Med 131:579-84

Vieth V, Schulz R, Heindel W et al (2018) Forensic age assessment by 3.0 T MRI of the knee: proposal of a new MRI classification of ossification stages. Eur Radiol 28:3255-3262

### Methodology

• prospective

• cross sectional study

• performed at one institution
